# Computer aided data acquisition tool for high-throughput phenotyping of plant populations

**DOI:** 10.1186/1746-4811-5-18

**Published:** 2009-12-10

**Authors:** Raju Naik Vankadavath, Appibhai Jakir Hussain, Reddaiah Bodanapu, Eros Kharshiing, Pinjari Osman Basha, Soni Gupta, Yellamaraju Sreelakshmi, Rameshwar Sharma

**Affiliations:** 1 School of Life Sciences, University of Hyderabad, Hyderabad 500 046, India; 2 JK AgriGenetics, Begumpet, Hyderabad 500016, India; 3 Department of Botany, St Edmund's College, Meghalaya 793003, India; 4 Department of Genetics and Genomics, Yogi Vemana University, Kadapa 516003, India

## Abstract

**Background:**

The data generated during a course of a biological experiment/study can be sometimes be massive and its management becomes quite critical for the success of the investigation undertaken. The accumulation and analysis of such large datasets often becomes tedious for biologists and lab technicians. Most of the current phenotype data acquisition management systems do not cater to the specialized needs of large-scale data analysis. The successful application of genomic tools/strategies to introduce desired traits in plants requires extensive and precise phenotyping of plant populations or gene bank material, thus necessitating an efficient data acquisition system.

**Results:**

Here we describe newly developed software "**PHENOME" **for high-throughput phenotyping, which allows researchers to accumulate, categorize, and manage large volume of phenotypic data. In this study, a large number of individual tomato plants were phenotyped with the "PHENOME" application using a Personal Digital Assistant (PDA) with built-in barcode scanner in concert with customized database specific for handling large populations.

**Conclusion:**

The phenotyping of large population of plants both in the laboratory and in the field is very efficiently managed using PDA. The data is transferred to a specialized database(s) where it can be further analyzed and catalogued. The "PHENOME" aids collection and analysis of data obtained in large-scale mutagenesis, assessing quantitative trait loci (QTLs), raising mapping population, sampling of several individuals in one or more ecological niches etc.

## Background

The precise documentation of phenotypic characters is of paramount importance for relating them to causative genes using currently available genomic tools/strategies of forward genetics or reverse genetics. A superior integration of high-throughput phenotyping technology and gene discovery that aims to unlock gene-phenotype relationships is a key step for better understanding of the genetic basis of such characters. Therefore, the development of a computable phenotypic database that allows rapid access to information on genes and associated phenotype(s) would extremely ease the process of data mining and data management. Since the representation of phenotypic information is a complicated process, there are few data standards for managing phenotypes and repositories even within a species [1]. In the process of associating phenotypes with genes, data integration plays a key role in correlating heterogeneous phenotypic data with genomic data at different levels. This is especially relevant in reverse genetics, where obvious morphological changes are rarely observed [2], or when mutations bring about only slight alterations in the phenotype. The technique of TILLING (**T**argeting **I**nduced **L**ocal **L**esions **IN G**enomes) [3,4] is a very powerful reverse genetics tool which is employed for high-throughput functional analysis. As with other reverse genetics strategies, this method requires extensive and precise documentation of material collected from which functional genomics data are produced [5]. However, one of the biggest challenges that are faced by such strategies is the availability of tools that would allow researchers to collect, access, organize, integrate and manage phenotypic databases across population(s), and which would enable the subsequent correlation of phenotypic information with genomic data. Thus, there is an urgent need for the development of technologies to encode and organize phenotypic information for high-throughput analyses [6].

Augmentation in capability of new hardware equipments that support mobile computing has opened a vast range of possible applications that in turn requires development of specific software(s) to run desired application. Palm PCs/Palm personal Digital Assistants (PDAs) are approximately one-fourth the size of notebook computers. Most of the mobile computer models receive user input from a virtual keyboard on screen, while others depend on an electronic pen and incorporate handwriting recognition. At present the hand-held computers/palm PDAs are embedded with the graffiti powered touch screen feature for user input. By writing with a stylus on the touch screen one is able to enter text/numbers, select options on the screen and give command to submit the data selected on the touch screen to the database present on PDAs. The most valuable feature available on current palm PDAs is the inbuilt barcode scanners, which makes the mobile computers/palm powered PCs/palm PDAs versatile equipment for the end user. Mobile computing has found applications in a vast range of commercial services right from airline baggage handling to tracking of international shipments of postal packets and is even emerging as a future replacement to the conventional data notebooks used in research laboratories. The recent releases of most software have built-in or add-on support for mobile computing, e.g. Wireless Internet Service provides Windows-CE users a stable link and flawless contact between compatible hand-held PCs/pocket PCs/Smartphone/palm-size PCs and the master PCs http://www.microsoft.com/windowsembedded/en-us/products/windowsce/default.mspx.

There are many compatible programming languages used in mobile computing. One of them is CASL (Compact Application Software Language) which is a prominent free programming language used for the development of software applications and is based on the legendary BASIC computer language for mobile computers/palm powered/palm PDAs/hand held PCs http://www.caslsoft.com. The vast implementation of the applications developed using CASLide software has made it the programmer's choice for developing new software for mobile computing. The mobile computing technologies in PDAs are used in diverse fields like census operations, courier services, surveys in marketing, eProbe studies in educational institutes, managing the stock in manufacturing plant, field studies, etc. One can also use these applications in fields like hospital clinical trials data management/human disease phenotype data management [1]. The necessity to implement unique identification to the individuals is a pivotal part in the management of these kinds of data. Advances in electronics and digital communications have contributed a revolutionary convention of unique identification procedure using "barcode" with various density and dimensions of 1D, 2D and 3D [7,8], giving the individuals a unique identity for the database management. In several spheres, barcoding is used for assigning a unique identity to the individuals for efficient database management [9] e.g. super market, transport department, hospitals, stock/inventory departments, biological and zoological institutes, etc. Here we describe customized phenotyping software developed in CASLide utilizing CASL programming language. This application was then loaded on to a PDA with an integrated barcode scanner for individual identification. With the ease of field data collection by PDA, we successfully phenotyped a large number of individual tomato plants belonging to an EMS-mutagenized population raised for TILLING.

## Results and Discussion

The present report highlights the use of personal digital assistants (PDAs) for collating a computable database of phenotypes of a mutagenized population of tomato (*Solanum lycopersicon *cv Arka Vikas). Moreover, the availability of touch-screen for data entry allows easy navigation to users. We used PDAs to collect phenotypic data from a heterogeneous population (~10,000) of tomato plants. Precise and absolute quantification of phenotypic data are indispensable for genetic analysis. However due to huge population sizes the simultaneous recording of multiple quantitative/qualitative characters most often becomes unmanageable. At the same time such huge quantity of data are crucially required for associating them with other data obtained experimentally in lab. The currently available technologies of the data management can be adapted for collating such phenotypic data, using the standard software(s) for data management systems [10] as described below for tomato.

### Phenotypic Catalogue (tomato mutant's phenotypic catalogue)

One of the foremost requirements prior to use of the electronic collection of phenotypic data is the selection of phenotypic parameters, which will be used for characterizing a selected population. Thereafter one needs to incorporate these parameters into a format recognizable by the application in use. In this study, we examined 15 different categories of plant morphological traits, which could be scored during different developmental stages of tomato beginning from germination to fruit ripening. The 15 categories selected encompassed the visually identifiable traits of the whole plant, leaf, flower, inflorescence, fruit and susceptibility to disease if any (Table 1). These categories were specifically selected to allow characterization of variations in the phenotypes within a large population of plants in a relatively short period with simple visual observations. For each of the 15 primary categories in our catalogue, there were one to seven subcategories describing the plant's phenotype in more specific details (Table 1). These parameters were selectively chosen from a wide range of tomato descriptors described by Menda *et al*., [11]. Using this system, the mutants were associated with a single subcategory, and thus the maximal number of attributes that each mutant can have equaled to the number of primary categories in the catalogue. This rationale is important for database queries in a web interface, which was designed to enable a straightforward search for simple traits as well as phenotypic traits affecting more than one attribute of the plant's morphology. Naturally, some mutant classes were described in the detail as their variations from the wild type were more pronounced under the field conditions.

**Table 1:** Tomato mutant's phenotypic catalogue

No.	Major Category	Sub-Category	No.	Major Category	Sub-Category
1	***Seed***	Germination	7	***Inflorescence***	Normal
		Seedling Lethality			Abnormal
			
		Slow Germination	8	***Flower Morphology***	Flower Homeotic Mutation
			
2	***Plant Size***	Extremely Small			Flower Organ Size
		Small Plant			Flower Organ Width
		Large Plant			Other Flower Morphology

3	***Plant Habit***	Inter-node Length	9	***Flower Color***	White Flower
		Branching			Pale Yellow Flower
		Aborted Growth			Strong Yellow Flower
			
		Other Plant Habit	10	***Fruit Size***	Small Fruit
			
4	***Leaf Morphology***	Leaf Width			Medium Fruit
		Leaf Size			Large Fruit
			
		Leaf Complexity	11	***Fruit Morphology***	Long Fruit
		Leaf Texture			Rounded Fruit

5	***Leaf Color***	Purple Leaf	12	***Fruit Color***	Yellow Fruit
		White Leaf			Orange Fruit
		Yellow Leaf			Dark Red Fruit
		Yellow-Green Leaf			Dark Green Fruit
		Dull Green/Gray Leaf			Green Fruit
			
		Dark Green Leaf	13	***Fruit Ripening***	Early Ripening
		Variegation			Late Ripening

6	***Flowering***	Early Flowering	14	***Sterility***	Partial Sterility
		Normal Flowering			Full Sterility
			
		Late Flowering	15	***Disease and Stress Response***	Necrosis
					Wilting

### PHENOME software for high-throughput phenotypic data collection

**Figure 1:**
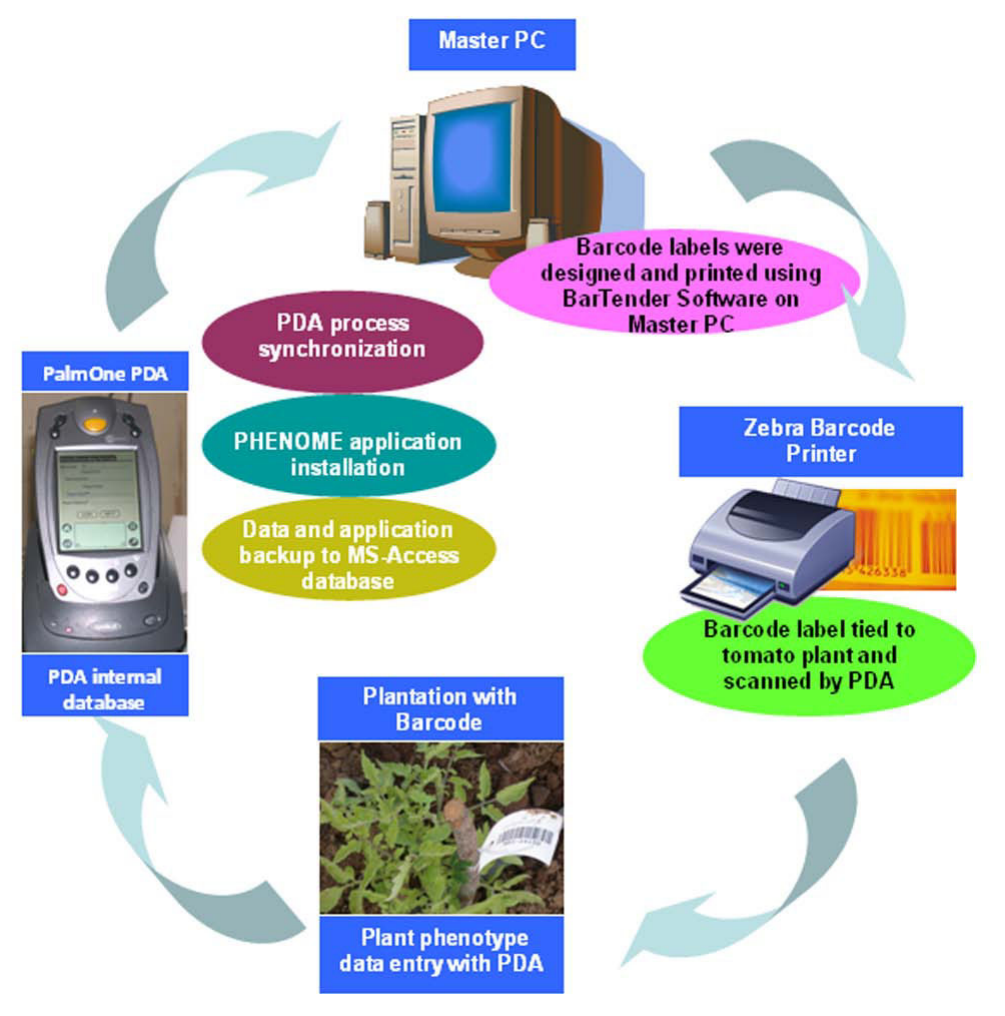
**The cycle of plant phenotype data collection using PDA with PHENOME software**. Plant ID barcode labels are tagged to plants. Using PDA phenotype data of plant is collected in the internal database of PDA. Once the PDA is synchronized with Master PC, the data from PDA database is synchronized with MS-Access database. The process to collect and synchronize the plant phenotype data using PDA from the field is cyclic process. For further changes in the plant phenotype catalogue, the modification has to be done in PHENOME software and it needs compilation and synchronization with PDA to update the changes (The process of modification in the PHENOME software is given in "PHENOME Read Me" file).

This software was developed to meet the needs of cataloging divergence in morphological traits for a large mutagenized tomato population that was raised for reverse genetics studies using TILLLING. We first tested the software by checking the accuracy of PDA barcode scanner to scan the barcode label tagged to the plants in the field. After that, we used PDA to record phenotypic data from a mapping population of tomato (~1,500 plants) and an EMS mutagenized cv. Arka Vikas TILLING population (~10,000 plants) and saved the collected data on to the Master-PC. To ascertain fidelity and ease of the data collection, we randomly cross-compared data collected using PDA with data collected manually. We found "PHENOME" to be a very convenient tool for data collection with the capability to accumulate phenotypic data rapidly and precisely with modest manual data entry. This accomplished by using data-handling technologies such as bar codes, personal digital assistants with inbuilt barcode scanner, touch screen, and computer system. The diagrammatic representation of the plant phenotype data collection using PDA is given in Figure 1. Using PDA, the plants were phenotyped at different stages of vegetative and reproductive growth using pre-defined parameters as enumerated in Table 1. Under field condition the barcode tag of each plant was scanned by a PDA equipped with an integrated laser scanner. Once the PDA recognizes the unique plant-ID barcode label it displays the predefined set of phenotypes for data entry. The visual observations of different parameters of plant phenotype were manually entered with a stylus by using drop down menu on the touch-screen of PDA.

**Figure 2:**
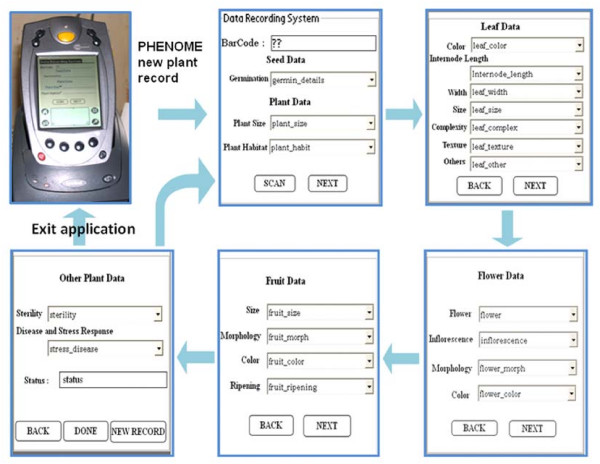
**The flow of interactive display of PHENOME application installed on PDA for user input**. Data is fed after barcode label is scanned by tapping the SCAN button using Stylus on the touch screen. The next or previous electronic forms will be displayed on the touch-screen by tapping on NEXT or BACK button. By tapping the DONE button on the last form, record is submitted in the PDA database.

PHENOME works in conjunction with a set of data entry forms for phenotype data collection. First, a "Data Recording System" form has to be filled which has an embedded SCAN button facility to record the barcode into the form, followed by "Leaf Data" form, "Flower Data" form, "Fruit Data" form and "other Plant Data" form as given in Figure 2.

**Figure 3:**
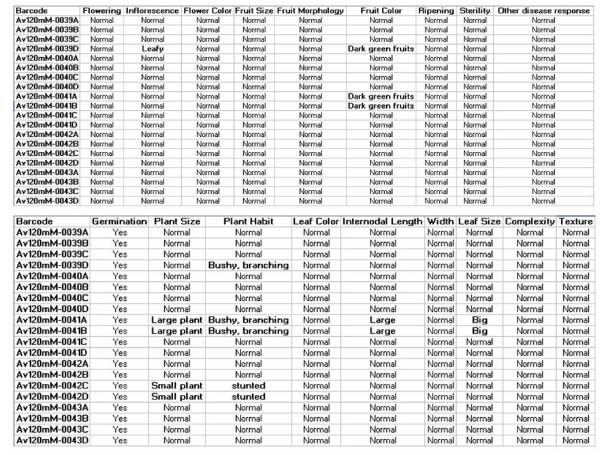
**The figure illustrates the representative output of phenotypic characters for few M_2 _plants**. The plant characters were recorded in the field using PDA and thereafter the data was transferred to the MS-Access database in Master PC. The distinguishing phenotypic characters observed are highlighted in green and red color.

At any given time, one can either continue the data record entry, or move to enter data for next plant by tapping the "NEW RECORD" button or tap on the ***home ***button on the PDA to exit the application. Thus, the PDA acts as an electronic organizer that enables an easy transfer of information from the field to a computer database and functions as an extension of the stored database. The data recorded in the field was transferred to the Master PC (Figure 3) by synchronizing the PDA. Once stored in the Master PC the data can be further classified and analyzed as per the need of the research program.

## Conclusion

The utility of the PHENOME application lies in its ability to characterize and evaluate very large sized populations. Accurate and efficient phenotypic characterization is one of the critical parameters for plant breeders for developing hybrids or cultivars that are superior to the existing varieties. The key step in phenotyping populations using a mobile technology is the plant descriptors (Phenotype catalogue) which have to be written in program code to be used in the PDA. We used CASL (Compact application solution language) for accomplishing this task. The CASL was selected for the development of data recording applications in PDA because of its free availability, ease of use, less memory consumption and feasibility for fast application development. Using this application, the plants were phenotyped at the different stages described using sets of predetermined characters. We used PHENOME to record and collate phenotypes of individual M2 line of an EMS mutagenized tomato population that was raised for setting up TILLING in tomato. In our experience, the use of PDA in the field allows efficient recording of large datasets in a short span of time for large number of plants. Similarly, the plant breeders, geneticists, ecologists and molecular biologists can utilize the PHENOME software to analyze large populations of plants. Though the PHENOME was developed to meet the need of phenotyping plants, with appropriate customization, the application can be used for collecting data from any other large population. Customization can be made for example in the plant descriptors or any other phenotypic descriptors required for any large population based on the requirement. This customization has to be implemented in the source code of PHENOME software i.e., project file, a file with a .CPJ extension, which is a PHENOME software core file encompassing all the required details for the application and the same has to be updated in the database file. Then the application could be used for collecting data from any other large population as modified in the source code. The PHENOME software can be edited to suit the end user's experimental requirements and demands. The PHENOME application system will enable collection of large datasets from the field thereby permitting efficient analyses and classification of information.

## Implementation

### Platform Technologies

The eight main components to develop "PHENOME" software were CASLide version 4.3 software http://www.caslsoft.com, PRCTools version 2.0 http://prc-tools.sourceforge.net/, CYGWIN version B.2.0 http://cygwin.com/, GCCTools http://cygwin.com, PalmOS SDK, ODBC (object database connectivity), PDA http://www.barcodediscount.com/catalog/symbol/spt1800.htm and PC (personal computer). To generate the barcodes "BarTender" software version 7.5.1 http://www.seagullscientific.com was installed on the PC and the barcode labels were printed using Zebra barcode printer (Zebra Technologies). The other remaining components used for the development/executing/synchronization processes of the software and the website addresses for the tools downloaded were mentioned in the PHENOME software package "PHENOMEReadMe" file. Most of the software components and tools are freely available under GNU Public License. The Microsoft Windows XP Operating System compatible software components were used which is user friendly to handle.

The core software language, CASL (Compact Application Solution Language) was used to develop the PHENOME software for PDA in CASLide v4.3 software (Integrated Development Environment). PRCTools component uses GCCTools to compile the project file and uses CYGWIN vB.2.0 Linux emulator software on Windows Operating System to make and generate the Palm OS (PDA Operating System) compatible p-code (pseudo-code) of the program for the PDA. The ODBC Connectivity on Microsoft Windows XP Operating System was created to synchronize the data between the remote device PDA and the database (Microsoft Access) on Master PC.

### Software Development

#### CASL and IDE Software

**Figure 4:**
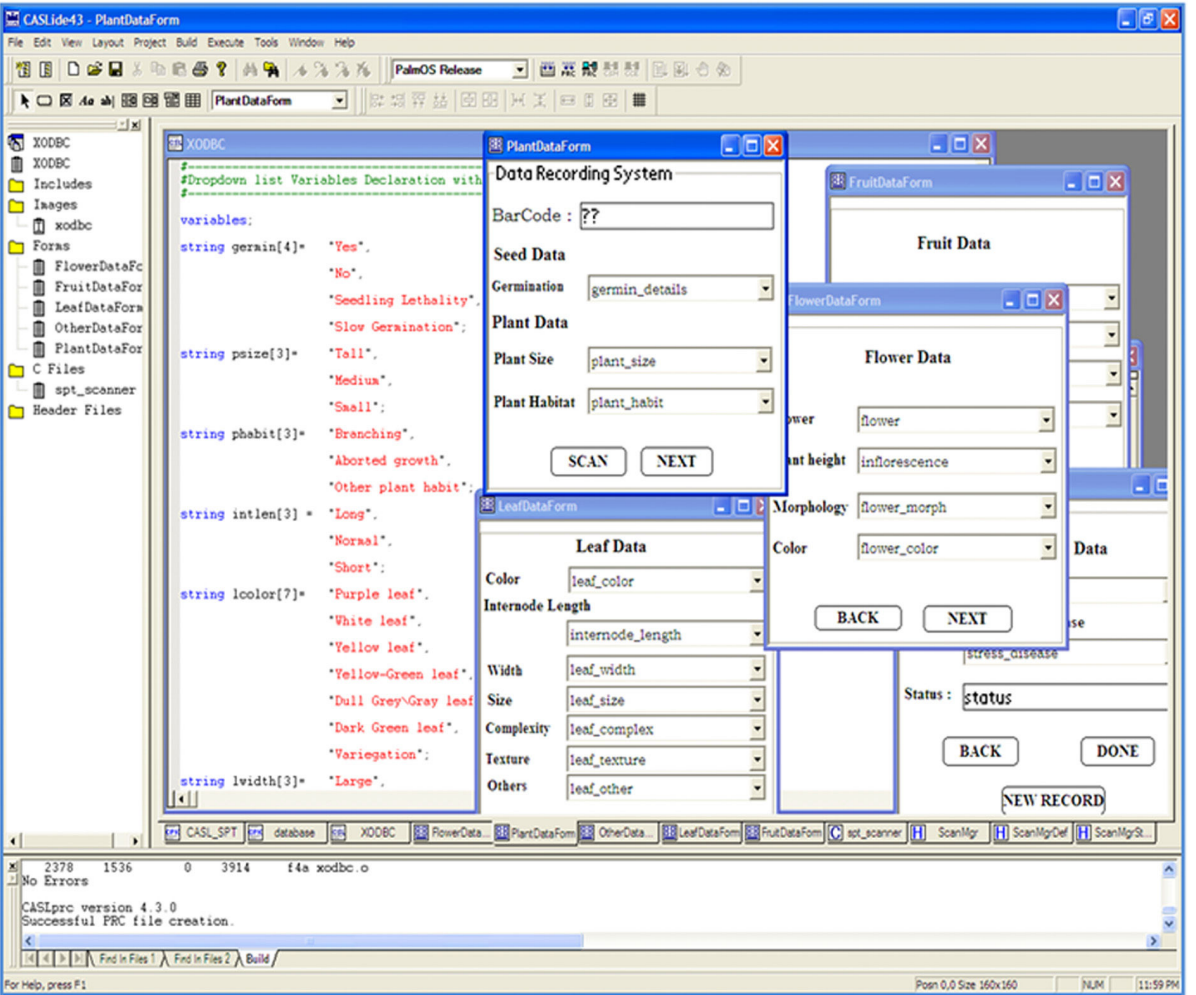
**CASL Integrated Development Environment of PHENOME software file**.

**Figure 5:**
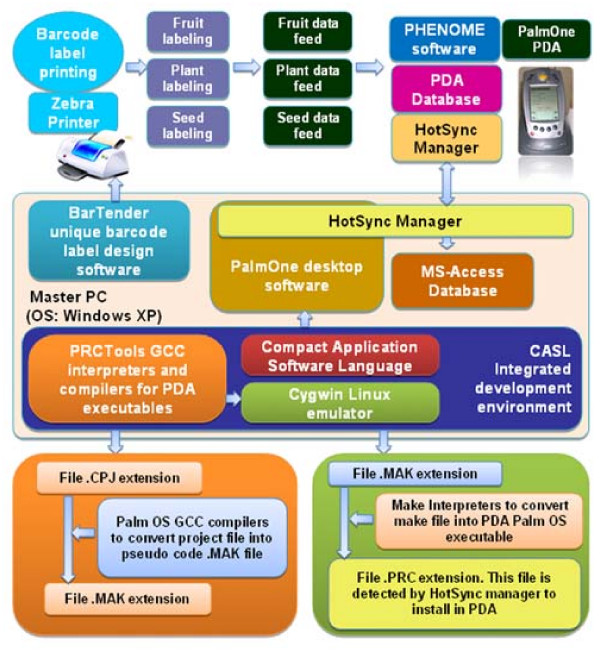
**Schematic representation of the PHENOME software**. PHENOME is installed on the Master PC along with the required software viz. CASLide V4.3, PRCTools, Cygwin, PalmOne Desktop and BarTender softwares. Barcode labels for Plant ID were printed using BarTender software for tagging the plant. PHENOME software was developed on CASLide software, the internal making for PHENOME was processed through PHENOME.CPJ project file to PHENOME.MAK make file using PRCTools software then PHENOME.MAK make file to PHENOME.PRC executable for PDA using CYGWIN software. PHENOME.PRC is installed on PDA using PalmOne Desktop software with HotSync manager. HotSync manager is also used to synchronize the data from PDA to MS-Access database on Master PC.

CASL is an event driven development tool e.g. objects are positioned on forms and when they are clicked, event occurs which is dependent on the precompiled CASL code that was created to intercept each event. The CASL language is similar to Visual Basics programming language (most popular GUI programming language of Microsoft) but unlike Visual Basics, it is freely available and it allows one to create programs for PocketPC/PalmPC/PDA taking advantage of the touch screen and inbuilt barcode reader available in PDA's. CASLide was used to design the electronic forms and for editing the programs code of PHENOME software. It is a swift application development environment for creating and deploying software to Palm and Pocket PC/Windows mobile devices (Figure 4):

Essentially, the program codes for the customized software, 'PHENOME', were written in file .CPJ extension using CASL language. These program codes were then compiled using PRC Tools (GCC and C++ compilers). PRC tools package is a collection of tools supporting C and C++ programming for Palm Operating System and compiles the program in file .MAK extension. Subsequently, CYGWIN was used to convert the compiled project file .MAK to file .PRC file, which are Palm OS's version of executable file. The file .PRC extension was then recognized by HotSync Manager of the PalmOne Desktop software for installing the complete software on PDA. The project files and data types used are described in details in sections below and the schematic representation of the PHENOME software is given in Figure 5.

#### Barcode label

The prerequisite for data collection in the field is the recognition of Plant ID on barcode labels. The Plant ID's were prepared in the MS-Excel sheet and this file was accessed using BarTender v7.5.1 Enterprise software for printing the unique barcodes on barcode labels for Plant ID's in the MS-Excel file. Unique plant-ID barcode label designed using BarTender software was printed on synthetic polyester, non-erasable, tear proof paper using laser printer. Each plant was tagged with these barcode labels, they were found to be resistant to rain and sunshine and even after six months of exposure to environment in open field, these could be read by the barcode scanning on PDA.

#### Project Files and Data Types

The PHENOME software was incorporated with *include *files, *c *files, *image *files, *form *files, *header *files and *project *files, six sub program files. Here *Include *files *"CASL_SPT" *and *"database" *possess barcode scanning feature and database creation on PDA. *CASL_SPT *file handles CASL interface definition to C functions to perform PDA Bar-Code scanner operations. The *database *file saves the phenotype data that was entered in electronic form on PDA. This helped to view, retrieve and modify the data records on PDA. The destination for PDA database on Master PC was MS-Access database. The database file name was incorporated in *database *include file of project as *database_source_name "CASLPlants"*;. The C file was *"spt_scanner"*, that holds the definition of barcode scanning properties; the form files are used for phenotype data collection in the application. We have created five forms *"PlantDataForm", "LeafDataForm", "FlowerDataForm", "FruitDataForm", and "Other DataForm" *to acquire the related data from individual plant. The library files were *"ScanMgr", "ScanMgrStruct" and "ScanMgrDef"*; these are barcode scanner supporting files of the application. The project file was file .CPJ extension, which is the core file of the project and gives all the information of the application needs; the *String *data type was used for storing the phenotype characteristic values. The *image files *contain the graphics used by PHENOME software to display user-convenient image variables for data recording system on PDA display screen, which supports only "Bitmap" image file types. Instead of plant descriptors, bitmap image files can be used to record the phenotypes, for example seed phenotype can be recorded against seed bitmap image. This PHENOME software package is versatile to include/remove the phenotype characteristic values e.g. for the phenotype character *"Germination"*, we were able to add new phenotype character value "Fast Germination" to the values existing ("yes", "no", "seedling lethality" and "slow germination") by modifying the program code respectively in the file .CPJ extension (In case any phenotype character/values is added/removed in the program code, the same should be created/deleted from the forms). The procedure for adding/removing the new values or characteristics is mentioned in "PHENOMEReadMe" file of PHENOME software package. PHENOME software does not support features like image capturing for the plants in the field using inbuilt camera on current releases of PDA's. However, this is also one of our long-term goals to develop our software for PDA's with built-in cameras, which include capturing the photos of the plants and subsequent annotations such as tags, captions, etc.

#### Data Recording System

Data recording forms were used to feed the plant phenotype data into PHENOME software on PDA using touch screen. The forms were designed by using a Form Control Toolbar in CASLide project by using objects such as buttons, labels, text boxes, list, drop down menu, etc. The display screen size of the form while developing the software has to be matched to the size of the PDA screen to prevent objects from being distorted; this was done in the menu project settings of CASLide v4.3 software before installing the PHENOME software on PDA. The default screen resolution of the Palm PDA was 160 pixels wide by 160 pixels tall. To facilitate the built-in barcode scanning feature in PHENOME software on PDA, a functionality program code was included in program files.

#### Creating PDA Executables

CASLide v4.3 software provides six execution modes: two debug and release modes for Windows, Pilot and PalmOS. After compiling the file .CPJ extension in Windows Release mode a .CSP file (Windows executable file) containing the compiled p-code was created and this file was used by the *CASLWin *interpreter to test the application in Windows environment before installing on PDA. After compiling the file .CPJ extension in PalmOS Release mode a file .PRC extension (PDA executable file) containing the PalmOS compatible compiled execution code (does not need any interpreter to intercept the code for running the software) was created and it executes directly, to display the PHENOME software on the PDA screen after the software was installed. The installation of file .PRC extension procedure was handled by HotSync manager (described in sections below) component. HotSync manager component installed the developed PHENOME software on the PDA and helped in synchronization between the PDA and Master PC.

#### ODBC Connectivity

Whenever remote devices (PDA) are connected to PC to transfer the remote data (e.g. phenotype data collected onto PDA using PHENOME software) onto the PC there should be PATH SET to synchronize the data between the remote device and PC. The database name mentioned in ODBC was same as in the program in the CASLide project database include file for database connection (data_source_name "CASLPlants";). The user may choose any of the output databases such as MySQL, Oracle, MS-Access, Excel, etc., for setting the path to the output database file where it is created on the Master PC. Alternatively, it can be done by connecting to the Data Base Management System (DBMS) directly and writing the database *user name *and *password *in program code. We used Microsoft Access database for the plant phenotype data collection.

#### HotSync Technology

The data collected in field on the mobile PDA was easily transferred to a Master PC by synchronising the PDA to the computer. To synchronize data, one must connect the PDA and Master PC directly with a cable or cradle. HotSync manager created automatic data backup every time it was synchronized [12]. The PDA output data was exported to MySQL Data Base Management System (DBMS). This DBMS was used to develop high-end Tomato Mutants Data Base currently being maintained at the Department of Plant Sciences, University of Hyderabad, Hyderabad, India. The conduit components of CASLide software were used for synchronizing PDA with the Master PC (Includes HotSync support for PalmOS and ActiveSync v3.7-4.x support for the Pocket PC/Windows Mobile).

## Availability and Requirement

PHENOME software is issued under GNU General Public License. Respective commercial software license is required viz. BarTender software for barcode label designing software (Seagull Inc.). PHENOME software is freely available from the authors upon request. It can be used, modified and distributed freely with prior acknowledgement from the original authors. The research projects benefited from PHENOME application should be cited in arising papers.

**Project name**: PHENOME

**Operating system(s)**: Windows XP or higher

**Programming language**: CASL

**Other requirements**: MS-Access 2003 or higher, MS-Excel 2003 or higher, PalmOne Desktop Software, Cygwin Linux emulator, CASLide 4.3 or higher, PRCTools compilers.

## Competing interests

The authors declare that they have no competing interests.

## Authors' contributions

RNV carried out the Software Development and Integration of database, AJH and RB, carried out tomato cultivation and designed requirements for phenotype data collection; AJH and EK assisted in software development and evaluation. RNV, YS, POB, SG were involved in evaluation of software and writing of paper. RS was involved in conceptualization and evaluation of software. All authors read and approved the final manuscript.
